# Pancreatitis Manifestation and Mimicking Gallstone Symptoms Caused by Fasciola Hepatica: A Case Report

**DOI:** 10.34172/mejdd.2025.435

**Published:** 2025-07-30

**Authors:** Zahra Bigdeli Shamloo, Ahmad Hormati, Amir Anushiravani

**Affiliations:** ^1^Department of Internal Medicine, Shariati Hospital, Tehran University of Medical Sciences Tehran, Iran

**Keywords:** Fasciola hepatica, ERCP, Pancreatitis, Case report

## Abstract

Fasciola hepatica is a parasitic trematode that infects humans, typically through the consumption of contaminated water or raw vegetables. The infection progresses through acute and chronic phases, with the latter often presenting as jaundice, cholangitis, pancreatitis, and cholecystitis due to bile duct obstruction. We report the case of a 53-year-old Iranian woman who experienced persistent eosinophilia, nausea, right upper quadrant and epigastric pain, and occasional chills for one year. Imaging revealed a dilated common bile duct (CBD) with sludge, and endoscopic retrograde cholangiopancreatography (ERCP) successfully identified and extracted five live Fasciola hepatica parasites. This case illustrates the vital role of early recognition and intervention in fascioliasis, as timely endoscopic management can prevent severe complications. Increased awareness among clinicians, especially in regions where the disease is uncommon, is essential to ensure prompt diagnosis and effective treatment of this often-overlooked infection.

## Introduction

 Fasciola hepatica is a leaf-shaped liver fluke that primarily infects livestock such as cattle and sheep, but can also cause disease in humans, particularly in endemic regions.^1^ Recent studies indicate that the prevalence of human infection with Fasciola hepatica in Iran ranges from approximately 1.7% to 2.5% across different regions, with some areas, such as Mazandaran, reporting a seroprevalence of 2.48%.^2,3^ Human infection occurs through the accidental ingestion of contaminated water or raw, unwashed vegetables harboring the infective metacercariae.^4^ The parasite’s lifecycle involves penetration of the intestinal wall and colonization of the biliary tree, leading to potential complications such as hepatitis, cirrhosis, and biliary obstruction.^5,6^ Fascioliasis manifests in two distinct phases: the acute phase and the chronic phase. The acute phase is characterized by the parasite penetrating the intestinal wall, migrating through the peritoneal cavity, and invading the liver capsule. This hepatic phase typically occurs 1-3 months after ingestion, during which the parasite measures approximately 5 mm in length. Patients may experience symptoms such as abdominal pain, fever, eosinophilia, and abnormal liver function tests. The chronic phase begins once the parasite enters the biliary ducts and matures.^7,8^ During this stage, Fasciola eggs are released into the stool, and the host’s immune system response undergoes modulation. Clinical manifestations in the chronic phase often include jaundice, cholangitis, pancreatitis, nausea, anorexia, and cholecystitis, primarily due to bile duct obstruction.^9-11^

 Additionally, anemia resulting from blood loss and iron deficiency can occur in both phases, caused by damage to the liver parenchyma during the acute phase and the biliary tree mucosa in the chronic phase. Notably, Fasciola hepatica infection can remain asymptomatic for extended periods, potentially years, making diagnosis challenging. Standard diagnostic techniques typically involve analyzing stool samples for the presence of eggs; however, these methods may exhibit low sensitivity, particularly in cases of early or mild infections. Serological tests, such as enzyme-linked immunosorbent assay (ELISA), enhance sensitivity and are especially beneficial during the acute phase, before egg production. Endoscopic retrograde cholangiopancreatography (ERCP) serves not only as an effective diagnostic method for visualizing the parasite in the common bile duct (CBD) but also offers a therapeutic option for the mechanical removal of the fluke.^12,13^ The primary treatment for fascioliasis is the antiparasitic agent triclabendazole, known for its high efficacy.^14^ In instances of resistance or intolerance to triclabendazole, alternative treatments like bithionol may be considered; however, these alternatives tend to be less effective and may lead to more adverse effects.^15^ Without appropriate treatment, fascioliasis can lead to recurrent cholangitis, secondary biliary cirrhosis, and potentially life-threatening complications.^16^

 Given the variable clinical presentation and the diagnostic challenges associated with fascioliasis, especially in endemic regions such as Iran, it is crucial for clinicians to maintain a high index of suspicion in patients presenting with hepatobiliary symptoms and relevant exposure history. The following case report highlights the complexities of diagnosis and management in a patient with fascioliasis, underscoring the importance of early recognition and intervention to prevent severe complications.

## Case Report

 A 53-year-old woman from Bostan Abad, Tabriz, was referred to the endoscopic ultrasound (EUS) center at Shariati Hospital due to persistent symptoms of nausea, right upper quadrant (RUQ) and epigastric pain intensified after meals, and occasional chills over the past year. Notably, she did not report symptoms such as pruritus, jaundice, anorexia, and weight loss.

 The patient had a history of hypertension and gastroesophageal reflux disease (GERD) type B, as confirmed by an upper gastrointestinal (GI) endoscopy. She was on medicines including losartan, bisoprolol, and esomeprazole.

 During the clinical examinations, her abdomen was fatty, soft, and non-distended. Mild tenderness was noted in the epigastric and RUQ regions, but no masses were palpable. The rest of her examinations were normal. Due to her symptoms of nausea and abdominal pain, an ultrasound (US) was performed to investigate the underlying cause.

###  Investigation and Imaging

**Figure 1:**
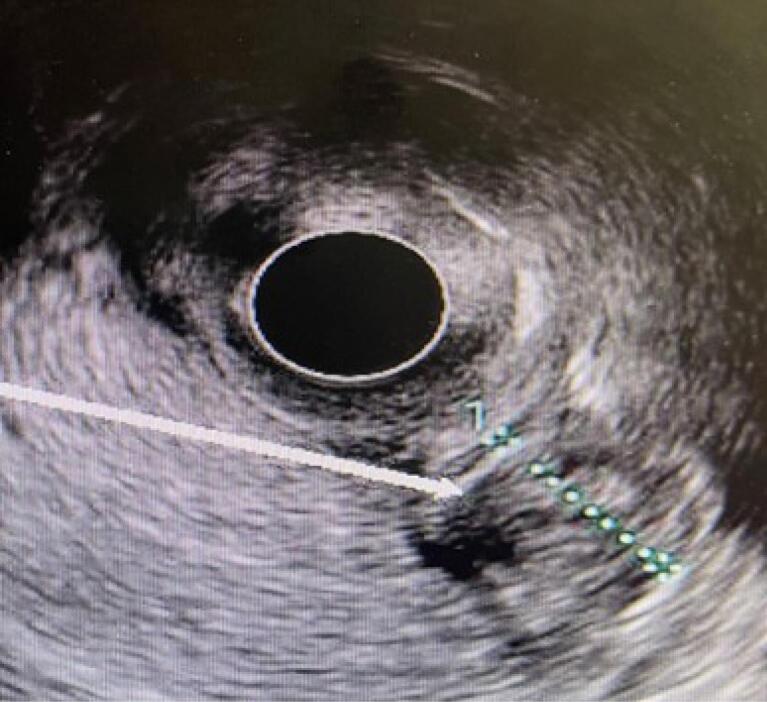
Endoscopic ultrasound (EUS) view of sludges

 The patient’s laboratory findings are summarized in Table 1. Sonographic findings revealed a liver span of 131 mm, a calcified granuloma measuring 5 mm in diameter in the right lobe of the liver, mild dilation of the intrahepatic bile duct, and mild wall thickening with significant sludge in the CBD, which was completely dilated to 14 mm. A porta hepatis lymph node measuring 15 × 8 mm was also observed. Further diagnostic evaluation with EUS confirmed a large amount of sludge in the distal part of the CBD (Figure 1).

**Table 1:** Laboratory data

**Marker **	**Value ** **(pre-treatment)**	**Value ** **(post-treatment)**
White blood cell (WBC) (10^9^/L)	14.93	6.83
Neutrophil (%)	78	57
Lymphocyte (%)	4	18
Monocyte (%)	6	11
Eosinophil(%)	12	14
Hemoglobin (g/dL)	12.5	10.8
Mean corpuscular volume (fL)	82.38	87.7
Creatinine (mg/dL)	0.8	0.72
Urea nitrogen (mg/dL)	8	4.7
ESR (mm/hr)	16	NA
CRP (mg/L)	13.5	NA
INR	1.14	
Alanine transaminase (ALT) (unit/L)	87	18.5
Aspartate transaminase (AST) (unit/L)	58	21.1
Alkaline phosphatase (ALK) (unit/L)	314	272.4
Total bilirubin (mg/dL)	1.4	0.4
Direct bilirubin (mg/dL)	0.6	0.2
Amylase (U/L)	1061	NA

**Figure 2:**
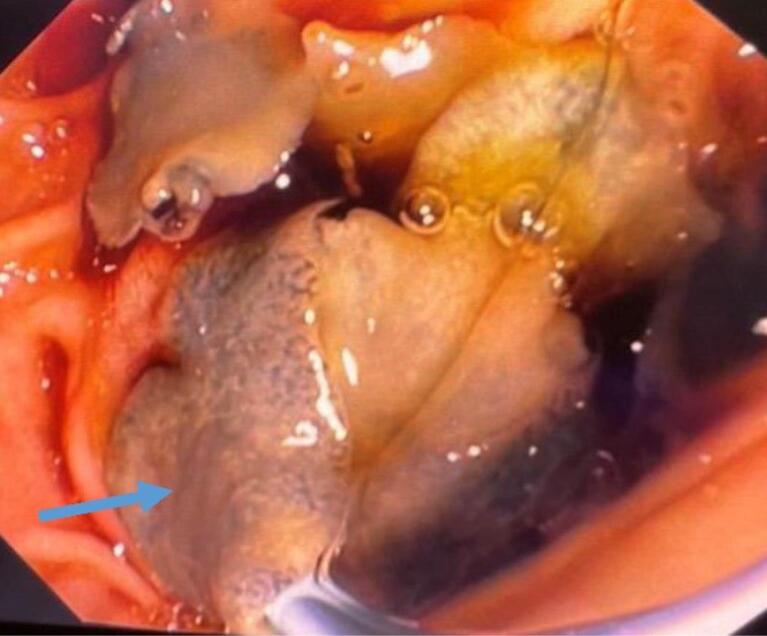
Endoscopic view of endoscopic retrograde cholangiopancreatography (ERCP). Removal of *Fasciola hepatica* by ERCP from the major papilla

**Figure 3:**
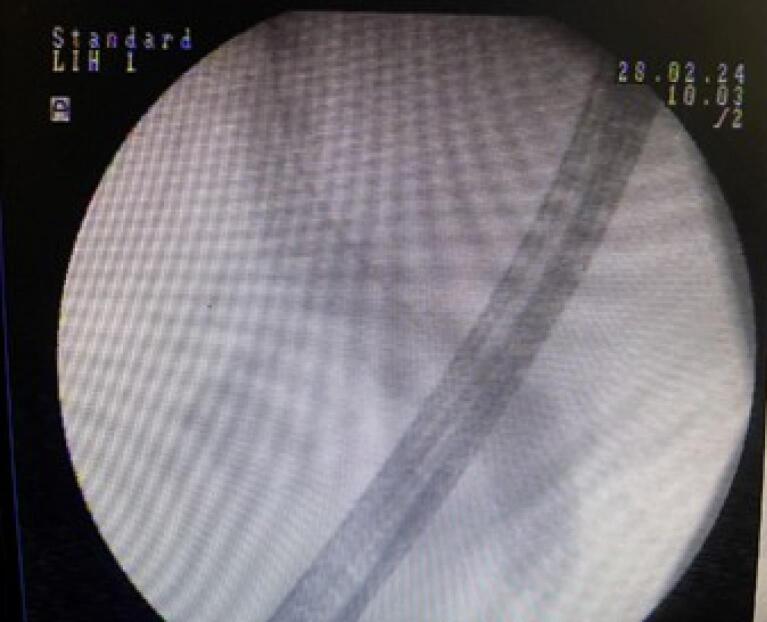
Fluoroscopic view of endoscopic retrograde cholangiopancreatography illustrating the common bile duct and common hepatic duct

**Figure 4:**
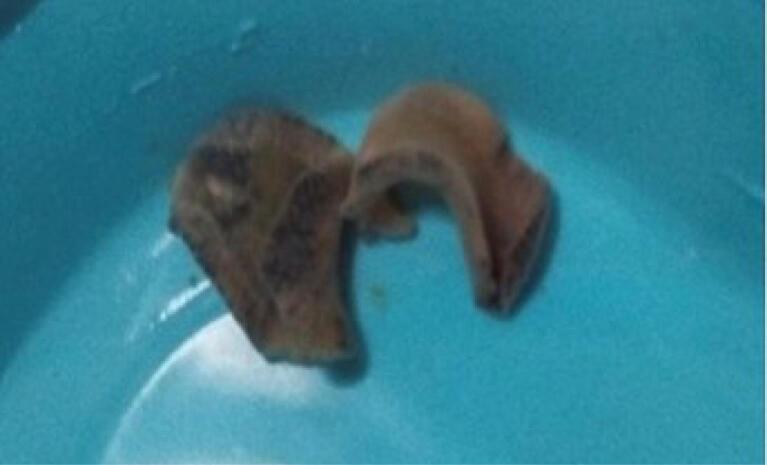
The extracted parasite by endoscopic retrograde cholangiopancreatography

 Based on the patient’s clinical symptoms, laboratory findings, and EUS results, we performed an ERCP. During the procedure, the major papilla was observed to be bulging. We performed selective cannulation of the CBD using a guidewire, sphincterotome, and needle knife. Contrast injection revealed a dilated CBD measuring 13 mm and a common hepatic duct (CHD) with a few defects measuring 7 mm in the CBD. A sphincterotomy was performed, followed by the use of a 12 mm balloon. The stone extraction balloon was used multiple times, resulting in the removal of sludge and five living leaf-like parasites (Figures 2 and 3). The parasites were morphologically identified as *Fasciola hepatica* (Figure 4). Finally, an occlusion cholangiogram showed clear results. After treatment, the patient showed significant clinical improvement. She was able to eat and drink without nausea or vomiting, and no longer complained of abdominal pain after meals. Only mild deep tenderness in the RUQ was noted, attributed to the recent ERCP procedure. Her temperature remained low without fever, and her vital signs were stable. She was discharged in good condition. The last follow-up was conducted at the time of discharge, as she did not return to our center for further evaluation.

## Discussion

 A 53-year-old female patient presented with chills, colicky abdominal pain, and nausea. Laboratory findings revealed hyper-eosinophilia (1791 eosinophils/μL) and mild hyperbilirubinemia (1.4 mg/ dL). EUS showed significant sludge in the CBD, prompting an ERCP for suspected choledocholithiasis. Following biliary sphincterotomy, a live, motile, flat-shaped worm measuring 25 × 10 mm was extracted from the CBD, identified as *Fasciola hepatica*. This case underscores the importance of considering fascioliasis in patients with biliary symptoms, especially those from endemic regions. Eosinophilia served as a crucial diagnostic clue. While imaging studies may suggest biliary parasitosis, ERCP proved valuable for both diagnosis and treatment through direct visualization and mechanical removal of the parasite.

 In patients presenting with RUQ pain, nausea, and biliary obstruction, common differential diagnoses such as gallstone pancreatitis and cholangitis must be carefully considered. Gallstone pancreatitis typically manifests with elevated pancreatic enzymes and imaging evidence of gallstones, while cholangitis often presents with fever, jaundice, and signs of infection. In this case, the absence of gallstones on ultrasound and EUS, combined with persistent eosinophilia - a hallmark of parasitic infections - raised suspicion of an alternative etiology. Imaging revealed biliary sludge and ductal dilation but no definitive stones, which, alongside the patient’s epidemiological background, supported the consideration of fascioliasis. Ultimately, ERCP provided definitive diagnosis and treatment by enabling direct visualization and extraction of live *Fasciola hepatica* flukes, underscoring its critical role in differentiating parasitic infections from other biliary pathologies.

 Our experience aligns with prior reports highlighting the utility of ERCP in managing biliary fascioliasis. For instance, one reported case involved a 13-year-old girl admitted with abdominal pain, nausea, and jaundice. The patient had leukocytosis and eosinophilia. The transaminase, bilirubin, amylase, and lipase values were elevated in the patient’s biochemistry. The abdominal ultrasound revealed dilatation and movement of hyperechogenic tubular structures in the intra- and extrahepatic bile ducts. ERCP successfully extracted three live, leaf-shaped *Fasciola hepatica* flukes, resulting in rapid clinical and biochemical improvement, with no recurrence observed during a one-year follow-up period.^13^

 Other published case series from Portugal, a region with low endemicity but rising imported cases, highlight the diverse clinical manifestations of biliary parasitosis. Three reported cases lacked typical risk factors related to environment, hygiene, or diet. The first two cases involved biliary colonization by *Fasciola hepatica*, with one patient presenting with obstructive jaundice and the other with recurrent pancreatitis. Abdominal US suggested biliary parasites in the first case, while cholangiography during ERCP revealed multiple small filling defects in the main duct, consistent with CBD dilation. After clearing the bile ducts using an extraction balloon, the diagnosis was confirmed as biliary parasitosis due to *Fasciola hepatica*. The third case involved a patient with acute cholangitis, where ERCP cholangiography showed elongated irregular filling defects and CBD dilation without intrahepatic duct dilation. Following sphincterotomy, the bile duct was cleared using a Dormia basket and balloon extractor, allowing the removal of Ascaris lumbricoides. All patients experienced significant clinical improvement shortly after ERCP and were discharged asymptomatic within 3 days of the procedure. ERCP played a crucial role in both diagnosis and treatment, with parasite removal followed by antiparasitic therapy. The success of ERCP, followed by anti-helminthic treatment (triclabendazole or albendazole), highlights its effectiveness in resolving acute symptoms and preventing long-term complications. The increasing prevalence of biliary parasitosis in non-endemic regions, driven by global travel and immigration, necessitates increased clinical awareness and a low threshold for suspicion. Although rare, these infections can lead to significant morbidity if overlooked, including recurrent cholangitis and secondary biliary cirrhosis. A history of travel to endemic areas should prompt consideration of parasitic infections, even in the absence of classic risk factors.^17^

## Conclusion

 This case highlights the diagnostic and therapeutic value of ERCP in managing biliary fascioliasis. Clinicians should maintain a high index of suspicion for *Fasciola hepatica* infection, especially in patients with relevant travel history, eosinophilia, and biliary symptoms, to ensure timely and appropriate intervention.
